# Supplementary material for: Enabling organ- and injury-specific nanocarrier targeting via surface-functionalized PEG-b-PPS micelles for acute kidney injury
Source: Nanoscale Horiz. 2025 Sep 26;10(12):3423–32. doi: 10.1039/d5nh00474h (PMC12498314; doi:10.1039/d5nh00474h)
Supplement: NH-010-D5NH00474H-s001 [file NH-010-D5NH00474H-s001.pdf]

Supplementary Information (SI) for Nanoscale Horizons.
This journal is © The Royal Society of Chemistry 2025

Supplementary Data

Table S1. PEG-*b*-PPS Micelle (MC) diblock copolymer.

	*fPEG**	MW (g/mol)
MeO-PEG_{45}-b-PPS_{18}-Bn	0.60	3,335

* Hydrophilic weight fraction of polymer.

Table S2. Targeting peptide constructs

*Construct**	*Lipid Anchor***	*PEG Spacer****		Peptide primary structure‡	MW (g/mol)	Purity‡‡
		Units	Length (Å)			
PG_{48}	PA	48	174.058	CLPVASC	3182	>95%
PG_{48}	PA	48	174.058	CYNTTTHRC	3588	>95%

* All peptide constructs are of the form [Lipid]-[PEG_X spacer]-[Cyclic peptide].
** Palmitoleic acid (PA).
*** The spacer length is presented as the number of PEG units.
The peptide construct with a 48-unit PEG spacer was synthesized using Fmoc-PEG24x2.
‡ Peptide primary structure is listed N-terminus to C-terminus.
‡‡ Peptide purity determined using LC-MS.

Supplemental Methods

SAXS modeling description

SAXS profiles were fitted in SasView 5.0.5 using the polymer micelle model following Pedersen (1999) [DOI:10.1107/S0021889899012248]. The model describes micelles as spherical cores with Gaussian polymer chain coronas, where the total form factor is given by:

$$P(q) = N^2\beta_s^2\Phi(qr)^2 + N\beta_c^2P_c(q) + 2N^2\beta_s\beta_cS_{sc}(q) + N(N-1)\beta_c^2S_{cc}(q) \quad (1)$$

Here, q is the momentum transfer vector $q = \left(\frac{4\pi}{\lambda}\right)\sin\theta$, N is the aggregation number of polymer chains per micelle, r is the radius of core, $\Phi(\mathrm{qr})$ is the normalized amplitude of the spherical core, and $P_c(q)$, $S_{sc}(q)$, and $S_{cc}(q)$ describes the scattering from the Gaussian polymer chains in the corona (the Debye function), core–corona cross interference term, and corona–corona interference term, respectively. The scattering contrasts are defined as

$$\beta_s = V_{core}(\rho_{core} - \rho_{solvent}) \quad (2)$$

$$\beta_c = V_{corona}(\rho_{corona} - \rho_{solvent}) \quad (3)$$

with V_{core} and V_{corona} the volumes and ρ the scattering length densities of each component. The amplitude of the spherical core is expressed as

$$\Phi(\mathrm{qr}) = \frac{\sin(qr) - qr\cos(qr)}{qr^3} \quad (4)$$

while the Debye function is

$$P_c(q) = \frac{2\,[\exp(-x) + x - 1]}{x^2},\ with\ x = q^2 R_g^2 \quad (5)$$

where R_g is the radius of gyration of the corona chains. The interference terms are given by

$$S_{sc}(q) = \Phi(qr)\frac{(1-\exp(-x))}{x}\frac{\sin(q(r + d\cdot R_g))}{q(r + d\cdot R_g)} \quad (6)$$

$$S_{cc}(q) = \left[\frac{(1-\exp(-x))}{x}\right]^2 \left[\frac{\sin(q(r + d\cdot R_g))}{q(r + d\cdot R_g)}\right]^2 \quad (7)$$

with d the penetration factor describing corona interpenetration.

The micelle diameter was calculated as $D = 2(r + d\cdot R_g)$. The DLS z-average was used to guide the initial core radius, which was refined during fitting, while R_g was varied to capture high-q scattering behavior. Scattering length densities (SLD) were treated as follows: the solvent SLD was fixed to the known value for PBS (~9.4×10^{-6}Å^{-2}), the core SLD was fixed across samples to the approximated value for PPS_{18} (9.1×10^{-6}Å^{-2}), and the corona SLD was left as the primary variable parameter, reflecting differences in peptide anchoring and hydration. All other model parameters were retained at default values. Parameter fitting was performed using the Levenberg–Marquardt algorithm, minimizing the chi-square (χ^2) function. A reduced χ^2 value close to 1 was taken as evidence of a satisfactory model fit.

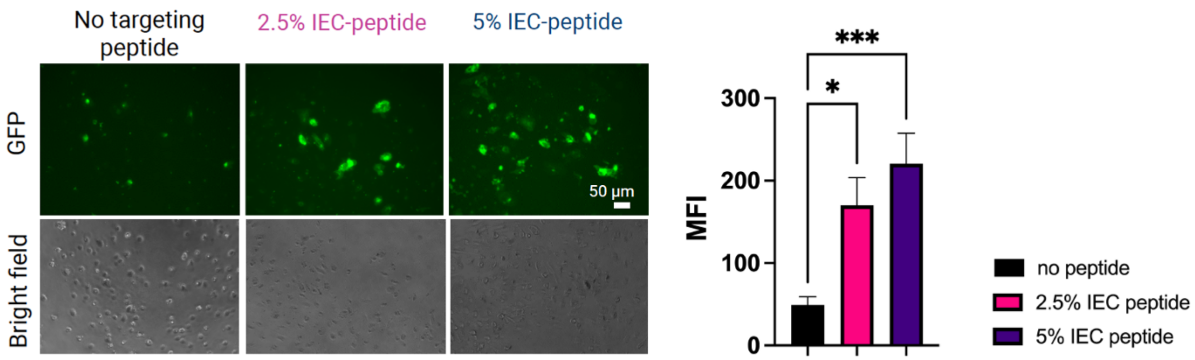

Figure S1. **MC functionalized with IEC-peptide demonstrate increased uptake by inflamed human endothelial cells**. Shown are representative images of the MC uptake across the indicated MC formulations. Median fluorescence intensity (MFI) was measured to quantify uptake. Statistics were determined using one-way ANOVA with Sidak correction for multiple comparisons. *, $P<0.05$; ***, $P<0.001$.
